# Influence of the sociodemographic profile of hunters on the knowledge and use of faunistic resources

**DOI:** 10.1186/s13002-022-00538-4

**Published:** 2022-05-15

**Authors:** Jeferson de M. Souza, Ernani M. F. Lins Neto, Felipe S. Ferreira

**Affiliations:** 1 Programa de Pós Graduação em Ecologia e Evolução, UEFS, Feira de Santana-BA, Brazil; 2 Núcleo de Estudos de Conservação da Caatinga (NECC)/Colegiado de Ecologia, UNIVASF, Senhor do Bonfim-BA, Brazil; 3 Programa de Pós Graduação em Ciências da Saúde e Biológicas, UNIVASF, Petrolina-PE, Brazil; 4 Programa de Pós Graduação em Ecologia Humana e Gestão Socioambiental, UNEB, Juazeiro-BA, Brazil

**Keywords:** Hunting, Vertebrates, Usage pattern, Cinegetic species

## Abstract

**Background:**

Hunting wild animals is essential for nutrition, clothing, predator control and disease treatment. As part of a system based on food choices and uses, it is influenced by ecological, economic and sociocultural patterns. In this context, the aim is to identify the game fauna of interest in the Brazilian semiarid region; indicate the methods, uses, patterns of choices and cultural importance of the fauna and identify which sociodemographic variables influence the knowledge and use of faunal resources.

**Methods:**

Information on hunting and fauna use was obtained through semi-structured interviews, complemented with free interviews and informal conversations. The cultural importance of the species was calculated through the current use value. The generalized linear model was created to verify whether the sociodemographic profile of hunters influences the knowledge and use of game species.

**Results:**

The results showed a representativeness of 56 species. The group of birds was the most representative in terms of taxonomic richness (48.2%), followed by the group of mammals (26.8%), reptiles (21.4%) and amphibians (3.6%). The animals mentioned are used for food, trade, control hunting (slaughter of animals considered invaders of property or harmful to humans), pets, zootherapy and ornamentation. Sociodemographic variables shaped the knowledge of faunal resources, in which the age of hunters showed a negative correlation with the number of known species.

**Conclusions:**

The meaning and forms of use attributed to each species depend on ecological, economic and sociocultural factors, which dictate the relationship between human communities and natural resources. Socioeconomic variables shape hunting patterns in all its aspects, whether in perception that hunters have of the resources, forms of use and utilization of hunting strategies.

## Background

Hunting is the main activity that allows human populations to interact with wild animals, being a material, moral and spiritual practice linked to socio-ecological systems. This activity is influenced by environmental, cultural and economic aspects that regulate the ways in which natural resources are used and therefore characterize a population, being fundamental for the physical and symbolic reproduction of different indigenous and local communities [1–5].

Hunted animals are used in various categories of use, being used essentially as food, but also as pets, commerce and in cultural presentations. Animal by-products from body parts are used for medicinal, religious, cosmetic, ornamental, tools and clothing purposes [6–10].

Hunting patterns related to the consumption preference of game fauna in an area depend on several intrinsic factors of each region, such as sociocultural aspects, land tenure situation, occupation, financial condition and preference for hunting [10–12]. It is assumed that when surveying the hunting profile of a community, it is possible to gather evidence that predicts hunting behavior in other locations and relate it to the socioeconomic conditions of hunters [13]. The literature has pointed out that local culture is an important predictor of knowledge about natural resources, especially plants and animals. At this point, ethnozoology contributes significantly to the understanding of the relationship between human beings and nature [14].

Identifying which cultural and social variables influence hunting allows establishing global patterns of which species are under greater pressure. The literature has pointed out that variables such as income, access to formal education, age and gender influence the use of faunal resources, and evaluating the action of these factors helps to understand the purpose of hunting and the categories of use related to faunal resources [11, 15, 16].

In the Northeast region of Brazil, hunting has been related to the subsistence and food security of rural populations, a factor reinforced by seasonal changes and difficulties arising from the Caatinga biome. However, the hunting phenomenon has proved to be complex, starting to present characteristics of commercial or sport hunting [9]. From this perspective, it is important to understand hunting methods and techniques, the patterns of fauna use and which variables influence this phenomenon. This knowledge is necessary for the formulation of public policies and the elaboration of projects aimed at sustainable hunting [17].

When designing projects and management plans for hunting control, it is recommended to consider the understanding of the social and economic realities of different populations [9], as the hunting profile is dynamic and may vary over time [13]. Thus, the objectives of this study are to indicate the game fauna in a municipality in the semi-arid region of Bahia; to identify the hunting methods, uses, choice pattern, cultural importance of the fauna and identify which sociodemographic variables influence the knowledge and use of the fauna. The hypothesis defended is that sociodemographic variables and cultural patterns linked to hunting influence the species that are known and used.

## Materials and methods

### Study area

**Fig. 1:**
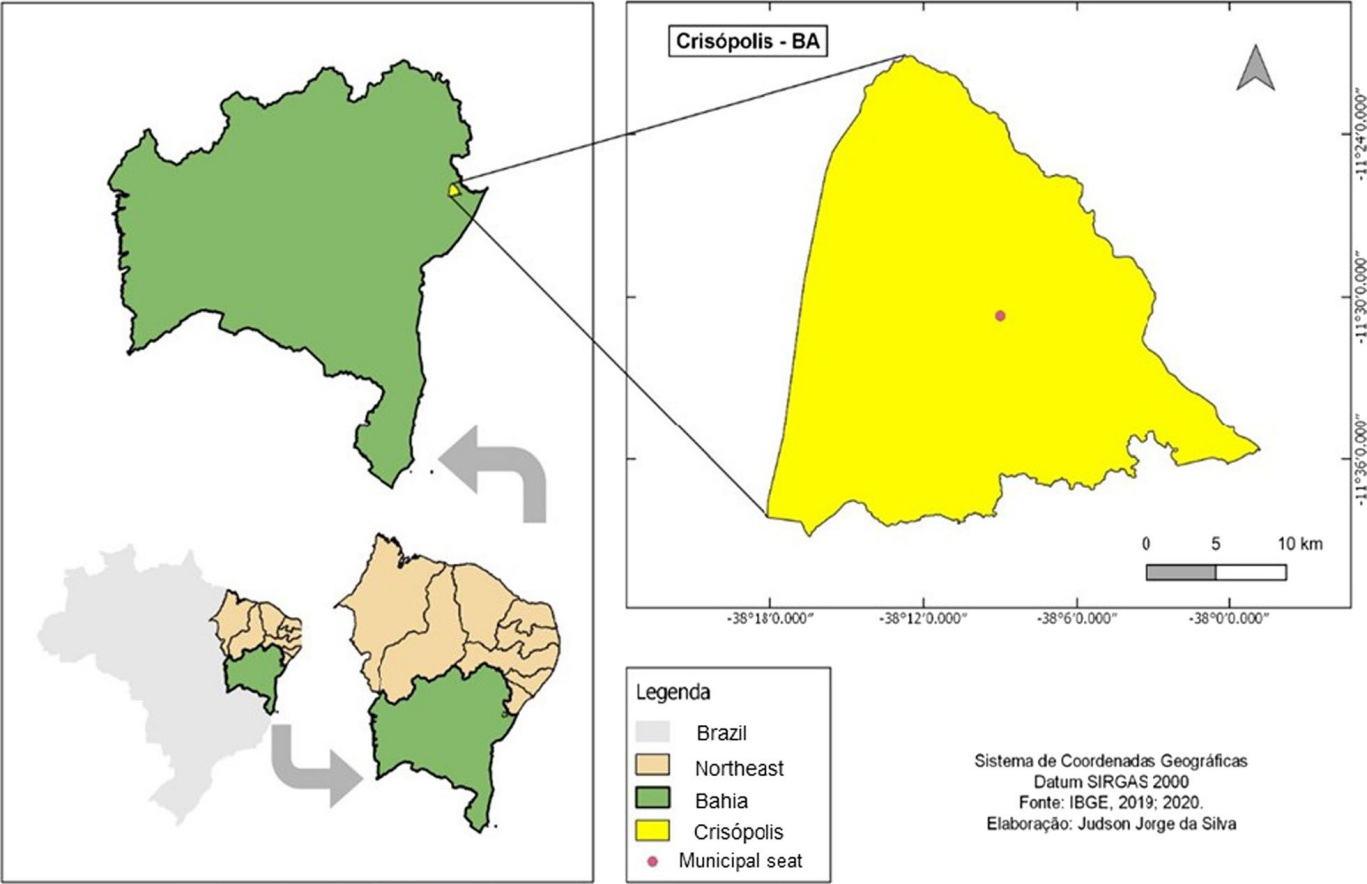
Map of the territory of the city of Crisópolis

The study was carried out in the city of Crisópolis (11° 30′ 39″ S 38° 09′ W), state of Bahia, 215 km from the capital, Salvador (Fig. 1). located on the coast of the mesoregion of Northeast Bahia and the microregion of Alagoinhas [18]. The predominant climate is semi-arid, characterized by low humidity, precipitation around 800 mm per year and average annual temperature between 25 and 30 °C, dividing the year into rainy and dry periods.

The municipality has a large extension of flat areas, characterizing the relief as a plateau. Its hydrography comes from tributaries of the São Francisco River, such as: Rio Itapicuru (bordering the municipalities of Itapicuru and Rio Real), Riacho Cabete and Marimbondo (bordering the municipality of Olindina), predominating the Caatinga, followed by some parts of the Cerrado.

The estimated population for 2021 is 21,219 inhabitants, of which 60% are rural residents [18]. The main source of income in rural areas is the cultivation of cassava, beans, corn, peanuts and sweet potatoes. Rural properties can be divided into small, medium and large. The large properties are represented by large-scale producing farms and the small and medium ones, predominant in the municipality, produce sustenance for the owner and his family, the surplus is sold in the city. In livestock there are cattle, goats and pigs. It is registered among residents of urban and rural areas the consumption and breeding of wild animals as a source of protein and income.

### Ethical and legal aspects

Participants were provided with information about the objectives and nature of the study, in case of acceptance to participate, they were asked to sign the Free and Informed Consent Term (ICF), which authorizes the collection, use and publication of the data obtained, as required of Resolution nº 196 of October 10, 1996, of the National Health Council. This study was submitted to the Ethics Committee of the Universidade Federal do Oeste da Bahia – UFOB, with approval under CAAE 50083520.4.0000.8060.

### Data collection

Data collection was conducted from December 2020 to March 2021. Information on hunting and game use was obtained through semi-structured forms, complemented with free interviews and informal conversations [19]. The forms were applied to people aged at least 18 years who had participated in at least one hunting event. Due to the conditions imposed by the SARS-CoV-2 pandemic, preventive measures were taken during the data collection stage, such as social distancing, mask use, alcohol gel and individual interviews.

The form dealt with questions about the socioeconomic profile of the participants (age, religion, monthly income, time of residence, profession, education, frequency of hunting, place of residence (urban or rural area) and family size), questions about the biology and ecology of hunted animals and cultural issues related to hunting (hunting motivations, forms of use, origin of knowledge and perception of informants regarding the use and protection of natural resources) [6]. The cited species were registered with their vernacular names, as mentioned by the participants and identified through comparison with the specialized literature. To avoid identification errors, photos of the cited species were shown to the hunters. To ascertain the conservation status of the species recorded, the List of Endangered Fauna of Bahia and the Red Book of Endangered Brazilian Fauna were used [17].

Sampling was non-probabilistic and intentional using informal conversations and the use of the “Snowball” technique [19]. To encourage respondents to remember more items, non-specific induction and re-reading of the free list were performed [20]. To maintain the confidentiality of the informant's identity, on occasions when their speeches were used, they were designated by the letter “C”, followed by the order in which they were interviewed (example: C1; C2; C3…).

### Data analysis

The data were analyzed in a quantitative and qualitative way, according to the model of union of the different individual competences, in which all the information related to the researched subject is considered [21]. To evaluate the sampling effort and verify if the number of interviews was significant in relation to the mentioned species, a species accumulation curve (*S*_obs_) was elaborated. To estimate game species richness, the first order nonparametric Jackknife estimator was used, which is based on the presence or citation of a species in a sample unit [19].

The analysis of species richness estimation was performed using the program EstimateS© version 9.1 [22], for data entry into the program, a matrix of the type interviewees (columns) × type of species (rows) was constructed in Excel. saved in text format (separated by tabs) and imported to perform the calculations. In the matrix, a value of 1 was assigned to species mentioned by the interviewee and 0 to those not mentioned. The values ​​obtained from the accumulation curve and the richness estimators, based on 1000 randomizations, were plotted on a graph, indicating the estimated species richness with a 95% confidence interval [17].

For the analysis of the cultural importance of the species, the Use Value (VU) was calculated. The VU estimates the relative importance of a natural resource, considering that the best-known resource is also the most used, and this relationship is directly proportional, that is, the greater the availability, the greater the use value of a resource [23, 24]. In this way, the current use value of each of the mentioned species was calculated, according to Rossato et al. [23] and adapted by Lucena et al. [25]. The formula used was

$$VU_{A} = \, \sum U_{A} /n;$$

where *VU*_*A*_ = Current value of species use (known and effectively used species); *U*_*A*_ = number of citations of current use by species; *n* = number of informants.

To test the hypothesis that sociodemographic variables and cultural patterns related to hunting influence knowledge about game species that are hunted and used, Shapiro-test was first applied to verify the normality of the data, and it was found that the data did not follow normal distribution.

Subsequently, a Generalized Linear Model (GLM) was created using the Poisson distribution. The number of species mentioned was used as a dependent variable and family income, family size, education, frequency of hunting, place of residence (urban or rural area) and age were used as independent variables.

The selection of the most parsimonious model followed through the elaboration of the global model using all the available variables and later the “StepAIC” function of the MASS package was used to eliminate the less significant variables, until reaching the model with the lowest AIC value (Information Criterion of Aikaike). The verification of residues was performed using the “rdiagnostic” function of the RT4Bio package.

Considering the heterogeneity of age (18 to 78 years old), the informants were divided into two groups: young (up to 40 years old) and experienced (from 40 years old) [26]. To identify whether there is a difference in the number of species cited between the two groups, the Mann–Whitney U test was used. The permutational multivariate analysis of variance (PERMANOVA) [27], was used to assess whether there was a difference in the composition of the cited species between the young and experienced groups.

Through the Jaccard distance with 1000 permutations, it was evaluated whether the number of techniques known by the informant, the biomass of the species, the categories of animal use and the reason for hunting influenced the composition of the species. Data tabulation, descriptive measures, construction of graphs and VU were performed in Microsoft Excel (2013). The Generalized Linear Model and the Mann–Whitney U Test were set in the R program version 4.1.0 [28], considering *p* < 0.05 as significant.

## Results

A total of 46 informants were interviewed (45 men and one woman). The age of the participants was heterogeneous, ranging from 18 to 78 years. All informants practice the Catholic religion and are mostly farmers (Table 1).

**Table 1:** Socioeconomic profile of participants (*n* = 46)

	Number of respondents and percentage
*Sex*	
Men	45 (0.98)
Women	1 (0.02)
*Age*	
Up to 29 years	17 (0.37)
30–39 years	8 (0.17)
40–49 years	7 (0.15)
50–59 years	5 (0.11)
60–69 years	8 (0.17)
More than 70 years	1 (0.02)
*Marital status*	
Married	34 (0.74)
Single	12 (0.26)
*Monthly income*	
≥ R$ 500,00	3 (0.07)
R$ 501,00 a R$ 1.100,00	36 (0.78)
R$ 1.101,00 a R$ 2.100,00	7 (0.15)
*Schooling*	
Illiterate	4 (0.09)
Elementary I Incomplete	17 (0.37)
Elementary I complete	5 (0.11)
Elementary II Incomplete	5 (0.11)
Elementary II Complete	1 (0.02)
Incomplete high school	2 (0.04)
Complete high school	12 (0.26)
*Profession*	
Farmer	44 (0.96)
Painter	02 (0.04)

The game fauna mentioned by the participants was represented by 56 species distributed in 34 families. It was not possible to identify three species (Araquã, Pato-d'água and Licuri-Chico) and one was identified only at the genus level (Rolinha, *Columbina* sp) (Table 2—h). Birds had the highest species richness (*n* = 27/48.2%), followed by mammals (*n* = 15/26.8%), reptiles (*n* = 12/21.4%) and amphibians (*n* = 2/3.6%). For avifauna, the families with the highest number of species cited were Thraupidae (*n* = 7), Columbidae (*n* = 4) and Tinamidae (*n* = 3). Among mammals, the most representative family was Dasypodidae (*n* = 3); and for reptiles the Colubridae family (*n* = 6).

**Table 2:** Game species used at the study site, use categories and current use values (VU)

Family/vernacular name/english name/scientific name	Category of use/interaction and number of citations								
	A	B	C	D	E	F	Total	VU	Status*
*Amphibians*									
Bufonidae									
Sapo/Frog—*Rhinella jimi* (Stevaux, 2002)		1					1	0.02	LC
Leptodactylidae									
Gia—*Leptodactylus vastus* Lutz, 1930	1						1	0.02	LC
*Birds*									
Accipitridae									
Gavião/Roadside-Hawk—*Rupornis magnirostris* (Gmelin, 1788)					2		2	0.04	LC
Columbidae									
Rolinha—*Columbina* sp.	16			1	1		18	0.39	LC
Juriti/Grey-fronted-Dove—*Leptotila rufaxilla* (Richard & Bernard, 1792)	13					1	14	0.30	LC
Asa branca/Picazuro-pigeon—*Patagioenas picazuro* (Temminck, 1813)	6						6	0.13	LC
Pocaçu/Pale-vented-Pigeon—*Patagioenas cayennensis* (Bonnaterre, 1792)	2						2	0.04	LC
Cardinalidae									
Azulão/Ultramarine-Crosbeak—*Cyanocompsa brissonii* (M.H.K. Lichtenstein, 1823)				2		1	3	0.07	LC
Falconidae									
Carcará/Southem-Caracara—*Caracara plancus* (Miller, 1777)					5		5	0.11	LC
Cracidae									
Jacu/White-browed-Guan—*Penelope jacucaca* Spix, 1825	2						2	0.04	VU
Thraupidae									
Sanhaço/Sayaxa-Tanager—*Thraupis sayaca* (Linnaeus, 1766)				2	1	1	4	0.09	LC
Colerinha/Double-collared-Seedeater—*Sporophila caerulescens* (Vieillot 1823)				7		1	8	0.17	LC
Cardeal/Red-cowled-Cardinal- *Paroaria dominicana* (Linnaeus, 1758)				19		4	23	0.50	LC
Papa-capim/ Yellon-bellied-Seedeater—*Sporophila nigricollis* (Vieillot 1823)				10		1	11	0.24	LC
Caboclinho/Copper-Seedeater—*Sporophila bouvreuil* (Müller, 1776)				6		1	7	0.15	LC
Canário/Saffron-Finch—*Sicalis flaveola* (Linnaeus 1766)				4		1	5	0.11	LC
Estevo/Green-winged-Saltator—*Saltator similis* d’Orbigny & lafresnaye, 1837				1			1	0.02	LC
Tinamidae									
Perdiz/Red-winged-Tinamou—*Rhynchotus rufescens* (Temminck, 1815)	17					1	18	0.39	LC
Codorniz/White-belied-Nothura—Nothura boraquira (Spix, 1825)	18						18	0.39	LC
Nambu/Spotted-Nothura—*Nothura maculosa* (Temminck, 1815)	13			1		2	16	0.35	LC
Tyrannidae									
Bem-te-vi/Great-Kiskadee—*Pitangus sulphuratus* (linnaeus, 1766)	1			3			4	0.09	LC
Passerellidae									
Tico-tico/Rufous-collared-Sparrow—*Zonotrichia capensis* (Müller, 1776)				1			1	0.02	LC
Icteridae									
Passaro-preto/Chopi-Blackbird—Gnorimopsar chopi (Vieillot, 1819)				6		2	8	0.17	LC
Cariamidae									
Siriema/Red-legged-Seriema—*Cariama cristata* (Linnaeus, 1766)	2			1			3	0.07	LC
Psittacidae									
Piriquito/Cactus-Parakeet—*Eupsittula cactorum* (Kuhl, 1820)				2			2	0.04	LC
Rallidae									
Galo-d’água/Purple-Gallinule—*Porphyrio Martinica* (Linnaeus, 1766)	6						6	0.13	LC
*Reptiles*									
Alligatoridae									
Jacaré/Broad-snouted-caiman—*Caiman latirostris* (Daudin, 1802)	6	3			2	1	12	0.26	LC
Boidae									
Jiboia—*Boa constrictor* Linnaeus, 1758	19	20			17		56	1.22	LC
Colubridae									
Cobra-coral-falsa/Brazilian-False-Coral-Snake—*Oxyrhopus trigeminus* Duméril, Bribon & Duméril, 1854					4		4	0.09	LC
Papa-pinto/Yellow-Rat-Snake—*Spilotes pullatus* (Linnaeus, 1758)					4		4	0.09	LC
Boipeba/Wagler’s-Snake—*Xenodon merremii* (Wagler, 1824)					1		1	0.02	LC
Cobra-cipó/Brown-vinesnake—*Oxybelis aeneus* (Wagler, 1824)					2		2	0.04	LC
Cobra-verde/Crown-Ground-Snake—*Erythrolamprus viridis* (Günther, 1862)					4		4	0.04	LC
Corre-campo/Green-Racer—*Philodryas nattereri* (Steindachner, 1870)					1		1	0.02	LC
Elapidae									
Cobra-coral-verdadeira/South-American-Coral-Snake—*Micrurus lemniscatus* (Linnaeus, 1758)		2			5		7	0.15	DD
Iguanidae									
Camaleão—*Iguana iguana* (linnaeus, 1758)	18	5			5	2	30	0.65	LC
Vipiridae									
Jararaca—*Bothropoides erythromelas* (Amaral, 1923)					1		1	0.02	LC
Teidae									
Teiú/*/*Giant-tegu—*Salvator merianae* Dumério & Bibron, 1839	35	21			3	2	61	1.33	LC
*Mammals*									
Canidae									
Raposa/Crab-eatin-Fox—*Cerdocyon thous* (Linnaeus, 1766)	17	14			18		49	1.07	LC
Caviidae									
Preá/Brazilian-Guinea-Pig—*Cavia aperea* Erxleben 1777	28				1	2	31	0.67	LC
Capivara/Capybara—*Hydrochoerus hydrochaeris* (Linnaeus, 1766)	6						6	0.13	LC
Cercidae									
Veado/South-American-Brown-Brocket—*Mazama gouazoubira* (G. Fisher [von waldheim], 1814)	8	2	1			1	12	0.28	LC
Cuniculidae									
Paca/Lowland Paca—*Cuniculus paca* (Linnaeus, 1766)	6					1	7	0.15	LC
Dasypodidae									
Tatu-peba/Six-banded-Armadilo—*Euphractus sexcinctus* (Linnaeus 1758)	35	8	5	4		5	57	1.24	LC
Tatu-verdadeiro/long-nosed-armadillo—*Dasypus novemcinctus* Linnaeus, 1758	24	4		2	1	5	36	0.78	LC
Tatuí/Seven-banded-Armadillo—*Dasypus septemcinctus* Linnaeus, 1758	19	2				1	22	0.48	LC
Dasyproctidae									
Cutia/Black-rumped-Agouti—*Dasyprocta prymnolopha* Wagler, 1831	2						2	0.04	LC
Didelphidae									
Saruê/White-eared-Opossum—*Didelphis albiventris* Lund, 1840	2				9		11	0.24	LC
Felidae									
Gato-do-mato/tiger-cat—*Leopardus tigrinus* (Schreber, 1775)	6				5		11	0.24	EN
Leporidae									
Coelho/Tapeti—*Sylvilagus brasiliensis* (Linnaeus, 1758)	16			2		2	20	0.43	LC
Molossidae									
Morcego/Palla’s-mastiff Bat—*Molossus molossus* (Pallas, 1766)					1		1	0.02	LC
Myrmecophagidae									
Tamanduá/Southem-Tamandua—*Tamandua tetradactyla* (Linnaeus, 1758)	2		1				3	0.07	LC
Procyonidae									
Guaxinim/Crab-eating-Raccon—*Procyon cancrivorus* (Cuvier, 1798)	2						2	0.04	LC
Unidentified species									
Araquã	6						6	0.13	
Pato d’água	4						4	0.09	
Licuri-chico				2			2	0.04	

Food (A); Zootherapy (B); Ornamentation (C); Estimation (D); Control (E); Trade (F)

*LC Least concern, *VU* vulnerable, *DD* insufficient data, *EM* in danger

**Fig. 2:**
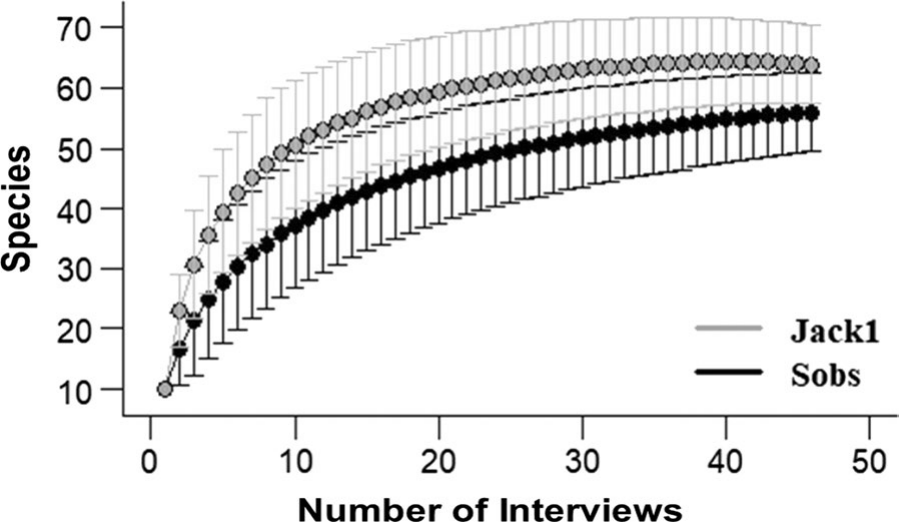
Species rarefaction curve, comparing the number of observed species (Sobs) and the estimated species richness (Jack1), generated from 1000 randomizations. 95% confidence interval

The Jack 1 estimator indicated a richness of 63.8 (± 3.22) species, presenting a value higher than the observed number (56 species, considering the 3 unidentified). The rarefaction curve shows stability in approximately 28 interviews, indicating sampling efficiency in data collection (Fig. 2).

The most cited species considering the use value were *Salvator merianae* (VU = 1.33), Armadillo, *Euphractus sexcinctus* (VU = 1.24); *Boa constrictor* VU = 1.22; *Cerdocyon thous* (VU = 1.07); *Dasypus novemcinctus* (VU = 0.78); *Cavia aperea* (VU = 0.67); *Iguana iguana* (VU = 0.65); *Dominican Paroaria* (VU = 0.50); *Dasypus septemcinctus* (VU = 0.48) and *Sylvilagus brasiliensis* (VU = 0.43). Regarding the conservation status, only the *Penelope jacucaca* and the *Leopardus tigrinus* have a vulnerable and endangered status, respectively.

**Fig. 3:**
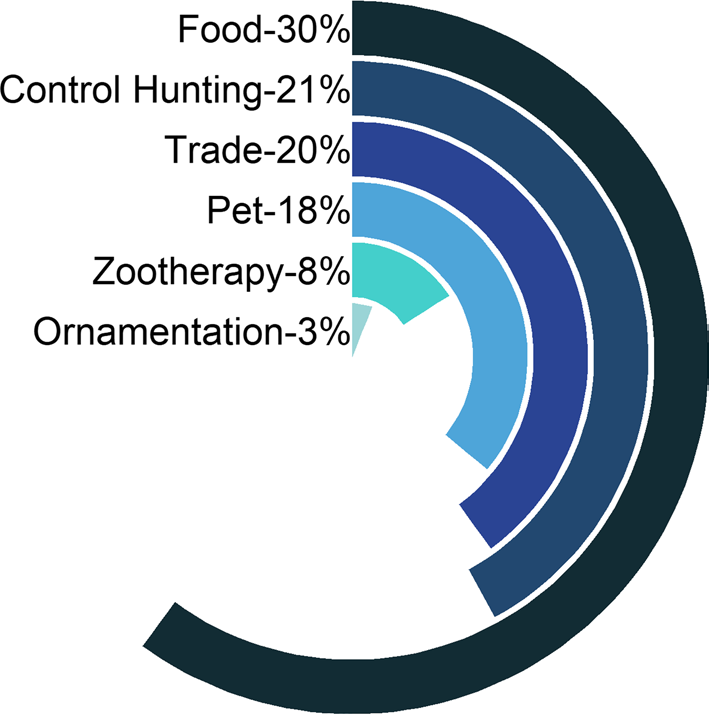
Percentage of citation for each use/interaction category

The mentioned vertebrates are used for food, medicinal use, ornamentation, pet, control and trade (Fig. 3). Of the species mentioned, 32 are used as food; 9 species as zootherapics; 3 species are used in ornamentation; 19 species are used as pets, which can be bred for the purpose of being slaughtered for consumption or can be marketed, as in the case of songbirds. Twenty-two animals are slaughtered by control hunting (slaughter of animals considered invaders of property or harmful to humans) and 21 game species are commercialized.

The species cited as medicinal resources were *Rhinella jimi, Caiman latirostris, Boa constrictor, Micrurus ibioboboca, Iguana iguana, Salvator merianae, Euphractus sexcinctus, Dasypus novemcinctus, Dasypus septemcinctus, Cerdocyon thous and Mazama gouazoubira*. The main parts cited for use in medicinal treatments were body fat, tail and leather.

Considering the distribution of taxonomic groups in the categories of use, it was found that birds and mammals are expressive as food resources, mammals and reptiles are of significant importance as zootherapeutics, the avifauna stands out in the pet and trade category, negative values associated with Reptiles became the group most killed by control hunting, due to being considered harmful or invasive on human properties.

Regarding the motivation to hunt, 27 (43%) participants stated that they practiced the activity for subsistence. However, leisure hunting also occupies a prominent place among the interviewed hunters, as 18 (29%) said they hunt for fun. It was found that there is a tendency to avoid the commercialization of game animals that are used as food resources, whether for practical or mystical reasons. Mystical reasons are associated with the belief that other people can use the bones of hunted animals in magical rituals that harm the hunter who sold the animal.

Regarding the motivation to use a certain species, 57% (*n* = 32) of the hunters mentioned that they kill or capture an animal considering the taste of the meat. The ease of commercialization ranks second as a motivation to capture a species, which is mostly representative of avifauna. In relation to hunting techniques and instruments, twelve techniques were mentioned, of which hunting with a dog and the use of a shotgun is highlighted, the badogue or slingshot is widely known, but not much used by more experienced hunters.

The model created was statistically significant, rejecting the null model, pointing out that sociodemographic factors and cultural patterns linked to hunting influence the number of known and used species. From the analysis of the most adjusted model (AIC = 271.63) it was found that only age, place of residence and hunting frequency are statistically significant, influencing the number of species mentioned. The other variables such as family income and family size were not significant (Table 3).

**Table 3:** GLM Poisson results, estimated parameters, standard errors, *z*-values and *p*-values

	Estimate	SE	*Z* value	Pr( >|*z*|)
(Intercept)	2.9454	0.2083	14.137	< 0.001***
Age	− 0.0132	0.0037	− 3.571	< 0.001***
Local (Ref = Urban)	− 0.1874	0.1093	− 1.713	0.08
Frequency (Ref = More than month)	− 0.4400	0.1622	− 2.713	0.006**
Frequency (Ref = Monthly)	− 0.1483	0.1682	− 0.882	0.37
Frequency (Ref = Weekly)	0.2515	0.1220	2.061	0.03*
Family Size	− 0.0397	0.0328	− 1.212	0.22
Family income	0.00006	0.00008	0.748	0.45

Signif. codes: 0 ‘***’ 0.001 ‘**’ 0.01 ‘*’ 0.05 ‘.’ 0.1 ‘’ 1

**Fig. 4:**
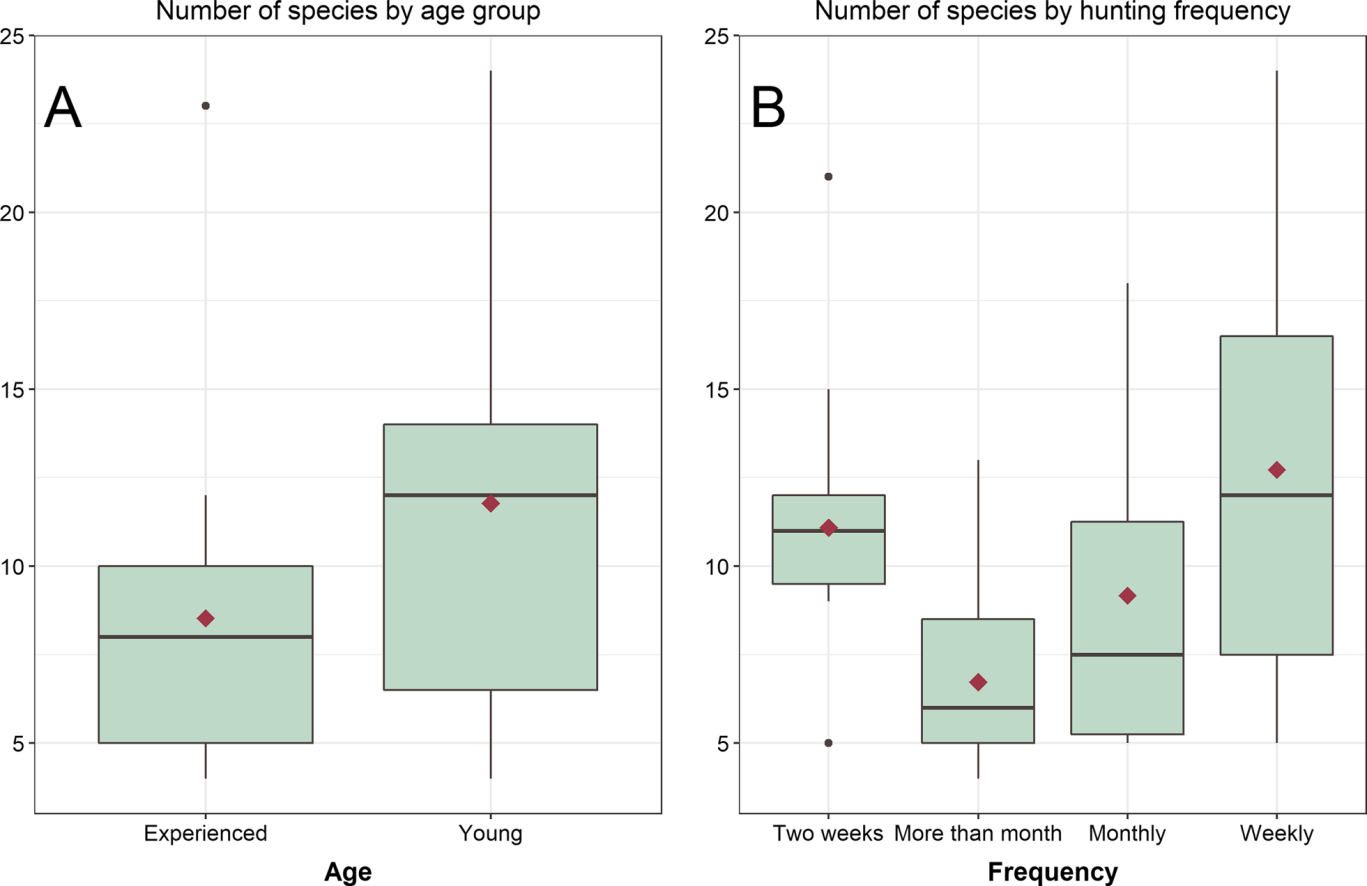
Comparison of the number of species cited by age group and hunting frequency. **A** Age groups; **B** Hunting frequency

The age variable showed a negative correlation, thus, with increasing age, the number of cited species is likely to decrease by 2% (OR 0.98; CI 0.97–0.99). When comparing the number of species mentioned by the two age groups, a significant difference was found (*U* = 166.5; *p* = 0.04), with young people (mean = 11.8; median = 12) citing more game species than the more experienced ones (mean = 8.5; median = 8) (Fig. 4A).

The place of residence significantly influenced the knowledge about the fauna, with participants living in rural areas citing an average = 11.12 ± 5.62, and hunters from urban areas having an average = 9 ± 5.15. Regarding periodicity, it was found that hunters who participate in hunting events more frequently cited a greater number of animals than those who hunt sporadically (Fig. 4B).

Thus, hunters who hunt for periods longer than one month showed a 36% decrease in the chances of citing game species (OR 0.64; CI = 0.46–0.88); those who hunt monthly have a decrease of 14% (OR 0.86; CI 0.61–119). On the other hand, hunters who go to hunting events weekly show a 28% increase in the chances of citing more species (OR 1.28; CI 1.01–1.63).

As for the composition of the species in the two age groups, it was found that there was no significant difference between the group of young and experienced hunters (df = 1; *R*^2^ = 0.023, *p* = 0.33). Through the PERMANOVA analysis, it was possible to verify that the difference between the age groups is not in the composition of the species, but in the number of citation of each species. The analysis also shows that young hunters cited songbirds more frequently than experienced hunters; on the other hand, animals used as food resources were more cited by older hunters.

## Discussion

### Usage patterns associated with faunal resources

The game fauna is a valuable resource for rural populations in several countries, in which the forms of use and the number of animals captured vary between populations, with numerous records in the literature of the various uses attributed to animals. In this study, birds had the highest taxonomic representation, followed by mammals and reptiles, the same result was found by Barbosa and Aguiar [6] and Santos et al. [9] in studies conducted in rural communities in the semi-arid region of Paraíba. Birds are most cited due to their greater abundance and diversity in the semiarid region compared to other vertebrate groups. The use of birds in animal trafficking or as pets also provides a greater number of interactions for this group [9, 29].

In a study with an Indigenous population in the northeast region, a greater preference for the consumption of poultry meat was found [30]. On the other hand, some authors point to a greater preference for mammals [31–33]. The most culturally important groups are mammals, birds and reptiles, and the use values ​​assigned to each of these groups depend on the objectives of the work developed and the type of interaction. It is worth mentioning that most publications on hunting are focused on interactions with mammals, which makes it difficult to compare the effect of hunting between taxonomic groups [34, 35].

Regarding the distribution of species in the categories of use, the results found are in accordance with the indicated in the literature. In this study, six categories of uses were identified, in which birds and mammals are most used as food; mammals and reptiles are more present in medicinal use; the avifauna is representative in the pet and trade categories and reptiles are the most slaughtered animals for control. The category of food use had the highest number of species cited, which corroborates other studies [9, 32, 36].

The preference for using mammals and birds as a food resource is a known pattern [2, 31]. It is assumed that mammals are preferable because of their greater body mass, resulting in a greater energy return, and birds due to species richness. Ethnobiological studies have pointed out the preference of hunters for large species following the logic of the Theory of Optimal Foraging, in which the energy gain is greater than the expenditure, at this point, variables such as body size and species abundance are present. As variables related to hunting [37, 38].

In the study developed by [24], for example, it was found that the preferred species are large animals, which have a good taste of meat and live in flocks, which makes hunting more productive. In another study that analyzed the relationship between the value in use and the biomass of mammals, it was found that the biomass did not influence the current use value of the mammals. In the Brazilian semiarid region, it is pointed out that there is no significant preference for large animals, since populations have developed strategies to take advantage of medium and small-sized species [8].

Still regarding mammals, Chaves et al. [20] analyzed the cost–benefit relationship, perceived abundance and taste preference, the results confirmed the predilections for large animals; also pointed out that more abundant species are ten times more likely to be hunted than rare species, and the preference for flavor increases the chances of a species being killed by 109%. Regarding wild birds, the preference for flavor is related to those species with a flavor close to chicken meat [2].

Based on the results of other ethnobiological studies, it was expected that vertebrate species would be widely used in the treatment of diseases, given that it is a widespread practice in Brazil, including in the Northeast region. The species cited as zootherapeutic in this study corroborate other results, since there are records of the use of animals in their entirety or fat for the treatment of diseases related to the respiratory and musculoskeletal system, especially swelling and inflammation [39, 40].

Although the highest number of citations for mammals is recurrent, reptiles have higher use values ​​in the zootherapy category [32]. In the semiarid region, the use of body fat from *S. merianae*, *B. constrictor* and *I. iguana* species cited in ethnozoological studies are generally used in more than one use category and when used for treatment they are indicated for more than one condition, recording up to 92 diseases or conditions in which animals are applied (whole or in parts), the main related conditions in the semiarid are wounds, rheumatism, thorn removal, earaches and sore throats [39, 41].

The avifauna is the group of vertebrates most involved with the trafficking of animals, being kept in captivity for their own consumption or commercialization. Birds of the Thraupidae and Icteridae families are used as pets due to their attractive colors, beautiful and pleasant song, the species of the Thraupidae family are the most illegally traded, while the Columbidae and Tinamidae families are preferred as a food resource because they have greater body mass [10].

The factors that favor the illegal trade of wild birds are availability, easy maintenance in captivity and ease of capture. Motivations such as entertainment and increased income are identified as influencers for the sale of birds. The value of birds depends on factors associated with the rarity or abundance of the species, the attractiveness of the song, the beauty of the plumage and companionship. Prices vary between species, studies indicate a variation from R$ 6.89 to R$ 1969.33 [42, 43].

Among mammals, the literature points out that the most commercialized species in the Caatinga biome are the giant armadillo (*Dasypus novemcinctus*) and giant armadillo (*Euphractus sexcinctus*), the sale usually occurs on a local scale, in which hunting is offered by hunters in the region or ordered by middlemen; the main motivation is the appreciation for the flavor of the meat [9].

Conflicting interactions between humans and non-human animals directly impact the fauna. It was found that the main target animals are carnivorous mammals, grassy birds and reptiles that are slaughtered for reasons related to appearance, fear and aversion. These results agree with what is pointed out in other ethnobiological studies. Conflicts arise when wild animals negatively impact human goals or when human goals interfere with wildlife needs [44].

The context of these conflicting relationships is different from hunting because the hunter aims at a product (e.g. meat, trophies, sports, medicines, etc.) for utilitarian purposes. In the slaughter of the animal due to the conflict relationship, the only objective is to eradicate the animal involved, being directed to predators in response to damage they cause to domestic animals, crops or for representing danger to people. In a review study it was found that the main mammal families involved in conflict in the world are Felidae, Canidae and Ursidae; among birds, hawks, hawks and vultures are protagonists; and snakes are the main representatives among reptiles [44, 45].

The aversions that lead to attitudes of persecution are related to characteristics of both the ecology and biology of animals and symbolic constructions of human culture. Some birds are persecuted because they emit sounds that are interpreted as creatures that bring bad luck, as an example of the shroud rips (*Tyto frucata*); the ability of the species to provoke feelings of disgust, repudiation and fear intensifies conflicts, and it is necessary to interpret these relationships and point out positive measures to mediate these interactions and promote the conservation of the species [16].

Subsistence hunting is widely documented, however, cases where the main motivation is leisure are portrayed [8]. In this study, hunters stated that the main reason for making use of an animal is taste, followed by ease of sale and abundance. The perspective around flavor has already been mentioned above, being a variable related to hunting, since the abundance of the species increases the chances of finding the resource and the energy returns [2, 3, 8, 20, 33].

Regarding the hunting methods and techniques mentioned in this study, other researchers had already described them. Hunting is started as a child, with the use of a slingshot and as experience is gained, the use of a shotgun and dogs is used, as methods that make hunting activity more efficient. Active techniques, in which the hunter actively moves behind the prey, represents a greater impact on the fauna, however, the use of passive techniques (traps) is preferable to capture small animals. The shotgun is the most cited instrument in hunting studies, being used both for protection and for killing animals. It is worth mentioning that, as found here, the same animal is hunted using more than one type of method or technique [2, 9].

### Influence of sociodemographic variables on the knowledge and use of fauna

Ethnozoological studies indicate that sociodemographic variables influence knowledge about the ecology of animals, use of faunal resources and patterns related to hunting [46]. Research indicates that the sociodemographic profile of the hunter can affect hunting efficiency [15, 47], number of species hunted [41], knowledge about resources [48], number of hunting techniques [12], perception of resource abundance [26] and predictors of conflicts between people and wild animals [43].

In this study, it was not possible to verify the difference in species knowledge between the genders of the hunters. However, it is necessary to discuss this variable considering the perspective of other studies. The literature points out that hunting is practiced by men, being common among traditional populations or communities linked to the countryside. It is worth mentioning that although hunting is traditionally linked to males, it is not restricted [39]. Archaeological findings have pointed out that this predominant man-hunter behavior is a recent cultural motivation, as ancestral hunter-gatherer communities encouraged contributions from all capable individuals, whether women, men or children [49].

In a case study on female involvement in hunting, women participate in many hunting activities around the world, whether encouraging hunting, performing rituals, tracking injured prey, preparing the hunt or supporting hunters. In the same study, the results showed that women assumed the role of hunter, however, they killed a lower diversity of species and achieved a lower hunting income when compared to men [50]. Regarding the differences in knowledge between men and women, it depends on each study developed, in the research by Santos [42], Lima et al. [31] there were no significant differences between the two groups; in another case study, men showed greater knowledge about species diversity and use value [48].

In this study, age was a statistically significant variable, but it presented a negative correlation, in which the group up to 40 years old cited more species than the group over 40 years of age, not corroborating other studies [15, 31, 48]. The age of the hunter has been a variable identified as statistically significant, presenting a positive correlation with the number of species mentioned. The literature suggests that older hunters are more efficient, know greater taxonomic diversity and master more techniques [11, 15, 26, 42, 47]. A different perspective is pointed out by both Lima et al. [31], and by Santos [48], in which age did not present a statistical correlation with the number of species.

The negative correlation of age with the number of species cited is explained by one of the specific processes of socioecological systems, which is memory. Memory is an often-overlooked variable, but it is important in the way people interact with nature and store information important for survival and reproduction. In this sense, memory influences the individual's local ecological knowledge, which reaches its peak in adulthood until middle age and maintains or decreases its knowledge as age advances [51].

The retrieval of memory about a person's knowledge is linked to temporal issues, that is, people more easily retrieve information related to recent memory of use [52]. This perspective is consistent with the context of the participants of this study, since the older informants are also those who practice hunting activity less frequently, therefore, they remembered a smaller number of game species than the participants who hunt more frequently. Frequency. In this sense, the periodicity variable showed a positive correlation with the number of species mentioned, which is justified by the idea that the lack of involvement with the environment makes people prone to losing knowledge about it [26].

Other sociodemographic variables have shaped interactions with fauna in several studies. Family size, for example, influenced hunting efficiency in an indigenous population, where hunters with smaller families hunted less [15], however, in another study, people with smaller families hunted more species [38]. Some studies have also demonstrated the relationship between hunting and income, and in some cases families with lower incomes hunt more to ensure food security [47] and in other cases there was no significant relationship with income [42].

It was found that there is no dissimilarity in the species composition between the age groups (Young and Experienced), however, the younger ones tend to hunt songbirds, aiming at commercialization with more recurrence and the experienced group hunt more animals for food. A distinct perspective is pointed out in a study developed with birds, in which young hunters hunted essentially for food, while older hunters presented a more versatile hunt, using birds for food, trade and pets [18].

## Conclusion

Hunting is the main hunting activity that allows humans to encounter wild animals, although food is the main motivation for the practice, it has been related to fun and leisure, in which hunters obtain faunal resources to consume in diverse ways. The meaning and forms of use attributed to each species depend on ecological, economic and sociocultural factors, which dictate the relationship between human communities and natural resources.

Socioeconomic variables shape hunting patterns in all its aspects, whether in the perception that hunters have of resources, forms of use and utilization of hunting strategies. In this sense, memory is a key component, in the sense that it allows individuals to remember and transmit their knowledge to other people, in which information related to recent memory of use is more easily retrieved. In addition, the discussions about how human beings create their socio-ecological systems and insert elements are far from being exhausted, and it is necessary to develop other studies to analyze the size of the effect of socially constructed variables (eg, food taboos, taste, animal appearance, belief and folklore) in the use of fauna.

## Data Availability

All data generated or analysed during this study are included in this published article.
